# Identification of loci controlling timing of stem elongation in red clover using genotyping by sequencing of pooled phenotypic extremes

**DOI:** 10.1007/s00438-022-01942-x

**Published:** 2022-08-24

**Authors:** Åshild Ergon, Øystein W. Milvang, Leif Skøt, Tom Ruttink

**Affiliations:** 1 Department of Plant Sciences, Faculty of Biosciences, Norwegian University of Life Sciences, P.O. Box 5003, N-1432 Ås, Norway; 2 Institute of Biological, Environmental and Rural Sciences, Aberystwyth University, Aberystwyth, UK; 3 Flanders Research Institute for Agriculture, Fisheries and Food (ILVO), Plant Sciences Unit, Caritasstraat 39, B-9090 Melle, Belgium

**Keywords:** *Trifolium pratense*, Flowering time, Haplotype, Pool-GBS, Selective genotyping, QTL

## Abstract

**Main conclusion:**

Through selective genotyping of pooled phenotypic extremes, we identified a number of loci and candidate genes putatively controlling timing of stem elongation in red clover.

**Abstract:**

We have identified candidate genes controlling the timing of stem elongation prior to flowering in red clover (*Trifolium pratense* L.). This trait is of ecological and agronomic significance, as it affects fitness, competitivity, climate adaptation, forage and seed yield, and forage quality. We genotyped replicate pools of phenotypically extreme individuals (early and late-elongating) within cultivar Lea using genotyping-by-sequencing in pools (pool-GBS). After calling and filtering SNPs and GBS locus haplotype polymorphisms, we estimated allele frequencies and searched for markers with significantly different allele frequencies in the two phenotypic groups using BayeScan, an F_ST_-based test utilizing replicate pools, and a test based on error variance of replicate pools. Of the three methods, BayeScan was the least stringent, and the error variance-based test the most stringent. Fifteen significant markers were identified in common by all three tests. The candidate genes flanking the markers include genes with potential roles in the vernalization, autonomous, and photoperiod regulation of floral transition, hormonal regulation of stem elongation, and cell growth. These results provide a first insight into the potential genes and mechanisms controlling transition to stem elongation in a perennial legume, which lays a foundation for further functional studies of the genetic determinants regulating this important trait.

**Supplementary Information:**

The online version contains supplementary material available at 10.1007/s00438-022-01942-x.

## Introduction

The timing of sexual reproductive development is an important agronomic attribute of all agricultural crops, and a trait with implications for fitness in natural populations. Proper timing in relation to the environmental conditions, in particular temperature and photoperiod, ensures a large and timely harvest of seed crops, but is also important in grassland species, in which the above-ground biomass is grazed or harvested for use as animal feed. For example, during the first stage of reproductive development of red clover (*Trifolium pratense* L.), the above-ground biomass increases fast. The timing is therefore decisive for the seasonal pattern of productivity, and in addition, it affects the competitive interactions with weeds and companion species. The timing also affects forage quality, as an increasing proportion of stem tissue reduces the digestibility. In spite of its importance in relation both to plant breeding and to fitness in natural ecosystems, there is very limited information available on the genetic control of the timing of transition to reproductive development in the large genus *Trifolium.* We therefore set out to identify genetic markers, chromosomal regions and candidates for genes and processes controlling timing of reproductive development in red clover, a perennial legume used extensively in production of silage and hay in temperate regions.

In its vegetative stage, red clover grows as a rosette consisting of many branches with non-elongating stems. The first visible sign of transition from vegetative to reproductive development is the initiation of stem elongation and appearance of internodes on some of the branches. This occurs in response to long photoperiods and increasing temperatures. There is little requirement for vernalization in red clover, but in some populations, the proportion of flowering plants increases after cold treatment (Fejer 1960; Van Dobben 1964). Under Nordic conditions, red clover of Nordic origin remains in a vegetative state throughout the year of sowing, while populations originating from further south can enter reproductive development. The reason for this difference is not known, but it might be due to a combination of juvenility, a facultative vernalization requirement and a very long photoperiod requirement in the Nordic populations. The transition from vegetative to reproductive development of the shoot apical meristem (SAM) and the onset of stem elongation are two components of sexual reproduction in rosette-forming dicots. Floral transition and stem elongation are usually coordinated processes, but stem elongation can be induced independently from floral transition (see examples in McKim 2020). Also, the two processes occur in different tissues in the shoot apex and may have slightly different responses to environmental variables (including red clover; Ergon et al. 2016).

*Trifolium* belongs to the vicioid/galegoid clade of the legume subfamily Papilionoideae, that also comprises the model species pea (*Pisum sativum*) and *Medicago truncatula* (Wojciechowski et al. 2004). Using sequences known from *Arabidopsis thaliana,* Hecht et al. (2005), Jung et al. (2012) and Kim et al. (2013) screened sequence databases for flowering-related genes of *M. truncatula, Lotus corniculatus* and *Glycine max.* They found that the majority of genes known from *A. thaliana* were present as orthologs in the legume genomes; some of the gene families appeared to have undergone differential expansion. Orthologs of some central genes (*CONSTANS (CO), FLOWERING LOCUS C (FLC)* and *FRIGIDA (FRI))* appeared to be missing, while orthologs of genes with similarity to *CO* and *FRI* were present. Putterill et al. (2013), Liew et al. (2014) and Weller and Ortega (2015) have reviewed what is known about the genetic control of flowering time in the legume family, which currently relates mainly to the photoperiod and vernalization/autonomous pathways. Compared to floral transition, the knowledge on the genetic control of stem elongation in rosette-forming dicots in general is limited (reviewed by Serrano-Mislata and Sablowski 2018; McKim 2019, 2020).

Red clover is an outbreeding species with a gametophytic self-incompatibility system (Taylor 1982). Thus, there is a considerable amount of genetic variation within cultivars, which are usually synthetic populations with a large number of parents. The genome size is estimated to be 420 Mb (Sato et al. 2005). Sequences of the red clover genome have been published by Ištvánek et al. (2014) and De Vega et al. (2015), but only the latter represents a draft genome at pseudo-chromosome level, covering 309 Mb of the genome (of which 164 Mb are placed on chromosomes). In a classical linkage map QTL study of red clover, Herrmann et al. (2006) identified eight QTLs for flowering time spread across all chromosomes except chromosome 1. In a recent study of a collection of 70 European and Asian natural populations and five modern cultivars by Jones et al. (2020), a genome-wide association study of flowering time identified one significant marker in a gene with homology to *VEG2*, a gene associated with flowering and inflorescence development in pea.

Our approach to advance our understanding of the timing of reproductive development in red clover was to identify individuals with extremely early or late stem elongation within one variety, sequence these in separate pools, identify polymorphisms, and search for genomic regions with significantly different allele frequency estimates in the two phenotypic groups (similar to a bulked segregant analysis). Sequencing pools of DNA from many individuals using reduced representation genotyping, e.g., genotyping-by-sequencing (GBS, Elshire et al. 2011) has emerged as an efficient means of estimating allele frequencies of a large number of loci in different types of (sub)populations (Dorant et al. 2019; Ergon et al. 2019). This is particularly useful for outbreeding species like red clover, where, particularly in a breeding context, it often is more relevant to characterize the genome at population or family level than on individual level. Sequencing of pools of individuals rather than separate individuals is a cost-effective way of obtaining accurate allele frequency estimates for a large number of markers and populations (Gautier et al. 2013; Byrne et al. 2013). In addition to single-nucleotide polymorphisms (SNPs), we utilized additional sequence polymorphisms that can be detected as variation in start and end points of the GBS read mapping positions (SMAPs, Stack Mapping Anchor Point polymorphisms), as well as short haplotypes defined by SNPs and SMAPs within reads (Schaumont et al. 2022). Single SNPs can be shared by several haplotype variants, and therefore be associated with more than one allele of nearby candidate genes. In such cases, allele frequencies may be aggregated at the SNP level, thus masking associations with the phenotype, whereas associations may be revealed by multi-allelic haplotype information (Rafalski 2002; Hamblin and Jannink 2011). Following SNP and haplotype calling, we employed three different methods to identify loci with significantly different allele frequencies in the two phenotypic groups; BayeScan (Foll and Gagiotti 2008), as well as two methods that utilize replicates of pools (Kawaguchi et al. 2018; Ergon et al. 2019), for stringent identification of genomic regions involved in regulation of the timing of reproductive development in red clover.

## Materials and methods

### Phenotyping and genotyping

Seeds of red clover (*Trifolium pratense* L., cultivar ‘Lea’ from Graminor AS) were sown in a greenhouse in September 2015 and grown at approximately 16 °C with a 20 h photoperiod (natural light supplied with 90 µmol m^−2^ s^−1^ PAR (HPQ/HTI-P lamps)). The number of days from sowing until the first elongating internode was 2 cm long (days to elongation, DTE) was recorded for each plant. The 52 earliest and 52 latest individuals (excluding non-elongating individuals) were selected for genotyping by pool-GBS. Pools were created as follows: DNA was extracted from leaf tissue of each individual with the DNeasy 96 Plant kit (Qiagen). The 52 individuals in each of the two phenotypic groups (early, late) were randomly divided into three subgroups of 17–18 individuals and equal amounts of DNA from each individual in each subgroup were combined in a pool, creating a total of six DNA pools. Each DNA pool was distributed into 15–16 wells per 96-well plate to create replicate GBS libraries. One plate each was used for *Pst*I and *Ape*KI single-digest GBS library preparation according to Elshire et al. (2011) and single-end sequenced on an Illumina HiSeq2000 instrument by Cornell University, Biotechnology Resource Center.

After demultiplexing, barcodes and 5’ restriction site remnants were removed, and reads were trimmed to maximum length of 74 bp (*Ape*KI) or 86 bp (*Pst*I). Reads were aligned with BWA-MEM (Li 2013) to the red clover reference genome sequence v2.1 (De Vega et al. 2015), in which 164 Mb ungapped sequence length out of a total estimated genome size of 420 Mb has been allocated to chromosomes. Alignments were sorted, indexed and filtered on mapping quality 20 (q20) with SAMtools 1.10 (Li et al. 2009). BAM files were converted to mpileup files with SAMtools and filtered to retain only genome positions with minimum read depth of 30, thus joining the neighboring GBS stacks and excluding the part of the genome without coverage. Mpileup files were used to calculate the Watterson’s theta estimator with NPStat v0.99 (Ferretti et al. 2013) with minor allele count equal to one read (MAC1), window-size equal to 10,000 bp, haploid sample size of 36, and maximum coverage equal to 8000. Per pool, a single genome-wide theta value was calculated as the mean across all windows (about 500 windows per sample). The NPStat derived theta values per pool-GBS sample were used as diversity prior for the Bayesian SNP calling algorithm implemented in SNAPE-pooled (Raineri et al. 2012) to identity significant SNPs in each pooled sample. SNAPE-pooled was run with settings:—priortype = informative, -fold = folded, -nchr = 36 for consistency with NPStat.

We used a custom python script to apply filters on the SNAPE-pooled reference allele frequency (RAF) data. Filters i–iv were applied in the following order and per sample: (i) SNP positions were deleted if the reference allele was not A, C, G, or T; (ii) SNP frequencies were set to missing data per sample when the two observed alleles were both different from the reference allele, or when the sum of the reference and the alternative allele read counts was lower than 30, (iii) using the Bayesian estimates of the probability of allele presence provided by SNAPE-pooled, we set the alternative allele frequency (and allele counts) to 0 if p(freq_alt_ ≠ 0) < 0.95 and the reference allele frequency (and allele counts) to 0 if p(freq_ref_ ≠ 0) < 0.95, and (iv) filtered out loci with low coverage (minimal read depth 27) that remained after removing read counts with filter iii; Next, we integrated all SNP frequency data into one matrix with six samples and all polymorphic loci, and applied filters v–vi: (v) we discarded SNP positions with more than two remaining alleles across the six samples per GBS library type; (vi) we retained only markers with an RAF between 0.05 and 0.95 (informative loci) in at least one sample, and frequency data in all six samples.

Next, haplotypes, of which there may be more than two variants per GBS locus, were called and their relative frequencies estimated using the SMAP package (Shaumont et al. 2022). The SMAP package is available on Gitlab (at https://gitlab.com/truttink/smap/) and a detailed description of the working procedure and guidelines are available online in the User Manual (https://ngs-smap.readthedocs.io/en/dev/home.html). In short, module *SMAP delineate* defines GBS loci by locating the outer positions of ‘stacked’ read mappings (called Stack Mapping Anchor Points (SMAPs)). *SMAP delineate* thus selects regions of the genome consistently covered by read mapping across the sample set, while simultaneously delineating start and end points for read-backed haplotyping of the SNPs identified with SNAPE-pooled (see above). The SMAP package further exploits polymorphisms in read mapping positions as additional information to define haplotype strings. A set of indexed BAM files with mapped reads, a custom BED file with GBS locus positions, and a VCF file with SNP positions then serve as input for the module *SMAP haplotype-sites*. *SMAP haplotype-sites* evaluate the read-reference alignment at each polymorphic position within a locus and creates a short haplotype string per read that combines the call of neighboring polymorphisms (SNPs and SMAPs) across the genome region covered by the GBS locus. It then counts the read depth per unique haplotype per locus, integrates all haplotype counts across all loci and samples, quantifies the relative abundance of haplotypes per locus and finally outputs the haplotype frequency table. Here, a GBS locus that contains multiple haplotype alleles is referred to as a haplotype polymorphism (HTP). *SMAP delineate* was run with parameters: mapping_orientation stranded, min_mapping_quality 30, min_cluster_length 50, max_cluster_length 130, min_stack_depth 5, max_stack_depth 1500, min_cluster_depth 30, max_cluster_depth 1500, max_stack_number 20, min_stack_depth_fraction 5, completeness 0, max_smap_number 20. A VCF file with SNP positions was used for haplotyping after filtering SNPs with data in at least one pool, only 2 alleles across all DNA-pool samples with data, and an RAF SNP frequency between 0.05 and 0.95 in at least one of the pools (see above). *SMAP haplotype-sites* was run with parameters: mapping_orientation stranded, partial include, mapping_quality 30, min_read_count 30, no-indels, min_haplotype_frequency 5 (meaning MAF > 0.05 in at least one of the DNA pools). After *SMAP haplotype-sites*, only loci with at least 30 reads (in total across all haplotype variants) in each of the six pools were retained, because downstream analyses required a complete genotype call matrix.

To visualize differentiation between the six pooled samples, a principal component analysis (PCA) was performed in The Unscrambler X v.10.3 (Camo Software, Norway), using minor allele frequencies (MAF) of all identified SNPs with a known chromosomal location.

### Identification of loci with significantly different allele frequencies in early versus late elongating groups

For each GBS dataset (*Pst*I and *Ape*KI), both SNPs and HTPs with significantly different allele frequencies in early and in late elongating pools were identified using three different methods; (i) an F_ST_-based test utilizing replicate pools (method 1, adapted from Ergon et al. 2019); (ii) a test based on error variance of replicate pools (method 2, adapted from Kawaguchi et al. 2018); and (iii) BayeScan v2.1 (Foll and Gaggiotti 2008). A previous study showed that two first principal components in a PCA of SNP data for 86 individuals derived from the same seed lot of cultivar ‘Lea’ as studied here explained only a few percent of the genetic variation (De Vega et al. 2015), suggesting that there is very little genetic structure and linkage disequilibrium. Hence, we did not consider it necessary to take population structure into consideration in the current analyses.

With method 1, for each SNP or HTP, allele frequencies in each of the six pools were compared with the average allele frequency in the three pools of the contrasting phenotype. Pairwise F_ST_ values $(\frac{\overline{{q}^{2}}-{\overline{q}}^{2}}{\overline{q}\left(1-\overline{q}\right)})$ were calculated, where $\overline{q}$ and $\overline{{q}^{2}}$ represent the weighted average of the allele frequencies or weighted squared allele frequencies in the pairwise comparison, respectively (when q was zero in both, F_ST_ was set to zero). This resulted in a total of six F_ST_ values for each SNP and HTP variant. A Chi-square test was used to identify significant *F*_ST_’s at different *P*-levels, using the test statistic *X*^2^ = *2NF*_*ST*_, where *2N* = the sum of genotyped gametes in the two populations (Hedrick 2011). Only SNPs and HTPs with a significant *F*_ST_ in all six comparisons, and a consistently higher allele frequency in early pools relative to the average of the late pools and vice versa, were regarded as significant at the given *P*-level. Corresponding estimates of the false discovery rate (*FDR*) were calculated for different *P*-levels as${\left(\frac{l*{P}^{3}}{d}\right)}^{2}$, where *l* is number of SNPs or haplotypes tested and *d* is the number of SNPs or haplotypes identified as significant, and a *P*-level corresponding to an *FDR* of 0.05 was chosen. The formula for calculating FDR is modified compared to the standard $(\frac{l*P}{d})$, because of the requirement of significance in all three replicate pools, and in both directions.

With method 2, a Chi-square test was used to identify SNPs and HTP variants with significantly different average allele frequency in early versus late elongating pools at different *P*-levels, using the test statistic *Z*^2^ = $\frac{{\left({\overline{q} }_{L}-{\overline{q} }_{E}\right)}^{2}}{{(V1}_{L}+{V1}_{E}+{V2}_{L}+{V2}_{E})}$, where $\overline{q }$ = the weighted average allele frequency across three replicate pools, *V1* = $\frac{\overline{q }(1-\overline{q })}{{2N}_{ind}}$, the variance due to sampling of the *N*_*ind*_ = 52 individuals in each phenotypic class, and *V2* = $\frac{{{\sigma }_{rep}}^{2}}{{N}_{rep}}$, the experimental variance between the *N*_*rep*_ = 3 replicate pools in each phenotypic class. Subscripts *L* and *E* refer to the late and early phenotypic classes, respectively. Here, V1 considers the variance due to the sampling of 52 individuals, and V2 considers the subsampling of 17 or 18 individuals out of 52, and all other experimental variance between (technical) replicates. Corresponding estimates of the false discovery rate (*FDR*) were calculated for different *P*-levels as $\frac{l*P}{d}$, where *l* is number of SNPs or haplotypes tested and *d* is the number of SNPs or haplotypes identified as significant, and a *P*-level corresponding to an *FDR* of 0.05 was chosen.

For the analysis with BayeScan, allele frequencies were converted into absolute allele numbers, using the number of haploid genomes that had been sequenced in each pool as the total number of alleles (34 for pools of 17 individuals, 36 for pools of 18 individuals). The allele numbers from the three replicate pools of each phenotypic group were summed, resulting in one value per marker (SNP or HTP) for both the early and the late group, which were analyzed using standard input parameters. BayeScan uses logistic regression to decompose locus-population F_ST_ values into population-specific and locus-specific components. Loci with different allele frequencies in the compared populations are identified as those, where the locus-specific component is necessary to explain the observed variation.

SNPs and HTPs identified by all three methods were retained for further description by searching for potential candidate genes in the 50 kb upstream and downstream genome region flanking each significant locus. Sequences and gene annotations were retrieved from the red clover genome sequence (Tp2.0, De Vega et al. 2015), using the Legume Information System (LIS, legumeinfo.org). The closest homologues in *A. thaliana* and *M. truncatula* were identified using coding sequences of the genes (CDS) as query in blastn searches (blast.ncbi.nlm.nih.gov).

## Results

### Phenotyping and genotyping

**Fig. 1:**
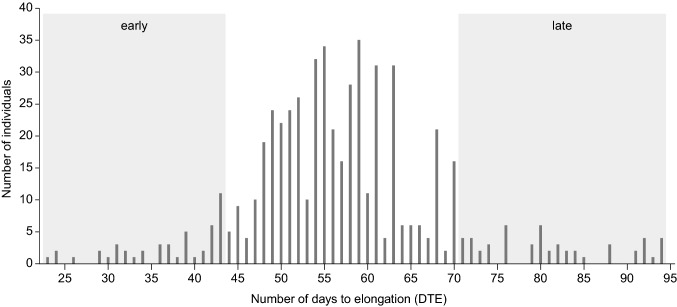
Timing of stem elongation (days to elongation—DTE) among 672 individuals of red clover, cultivar “Lea”. Hundred and forty-six individuals did not start stem elongation during the 95 days of the experiment and are not shown. The shaded areas at each end of the histogram show the groups of individuals that were selected for genotyping

The number of days to stem elongation (DTE) varied from 23 to 94 (Fig. 1). Of the 672 plants tested, 146 plants did not elongate during the 94-day course of the experiment and were excluded. Fifty two of the earliest elongating individuals (average DTE of 37.7, range 23–43, top 10%) were randomly assigned to one of three replicate early pools, and similarly, 52 of the latest elongating individuals (average DTE of 80.5, range 71–94, bottom 10%) were randomly assigned to one of three replicate late pools.

The six pools were genotyped by pool-GBS and a total of 229 M and 327 M high-quality reads were obtained for the *Pst*I and *Ape*KI libraries, respectively. After SNP calling and further filtering, we obtained 12,074 and 91,136 SNPs with read depth > 30 in each of the six pools and MAF > 0.05 in at least one of the six pools from the *Pst*I and *Ape*KI libraries, respectively (Supplementary File 1). Of these, 66,458 SNPs (64%) had a known chromosomal location, the rest were located on unplaced scaffolds. Haplotype calling and filtering (read depth > 30 in each sample, and haplotype MAF > 0.05 in at least one pool) resulted in 4653 HTPs with 2–13 haplotype variants per HTP locus (3.6 on average) based on the *Pst*I libraries, and 41,073 HTPs with 2–20 haplotype variants per HTP locus (3.6 on average) based on the *Ape*KI libraries. Of these, 30,215 (66%) had a known chromosomal location. For the combined *Pst*I and *Ape*KI libraries, this equals an average density of one HTP with known chromosomal location per 5.4 kb of the chromosome-anchored part of the reference genome sequence.

**Fig. 2:**
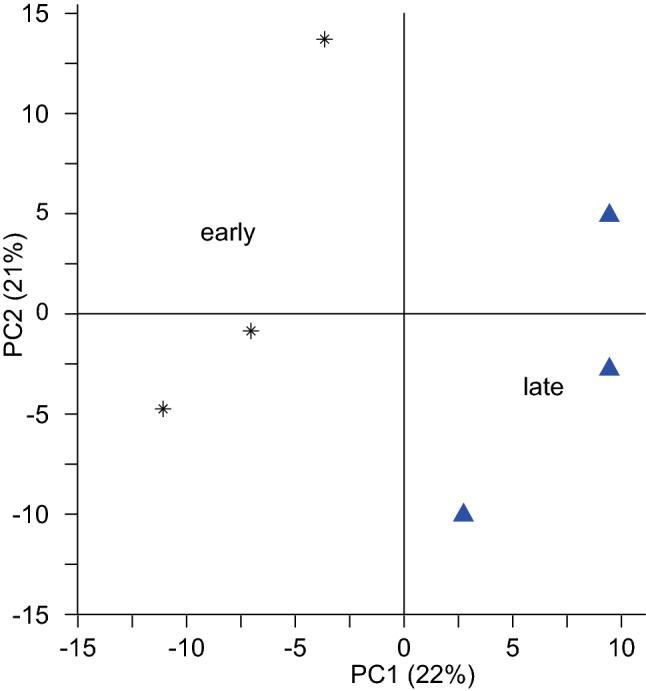
Principal component analysis of minor allele frequencies of 66,458 chromosomal SNPs in three early (stars) and three late elongating (triangles) pools, each consisting of equal amounts of DNA from each of 17–18 red clover individuals

A PCA of MAF data for the 66,458 chromosome-anchored SNPs showed that there was a large amount of random variation in allele frequencies among the replicate pools. However, the first principal component, explaining 22% of the variation, separated the three early pools from the three late pools (Fig. 2), indicating that variation between replicate pools is smaller than variation between early and late pools.

### Identification of loci with significantly different allele frequencies in early versus late elongating groups

**Fig. 3:**
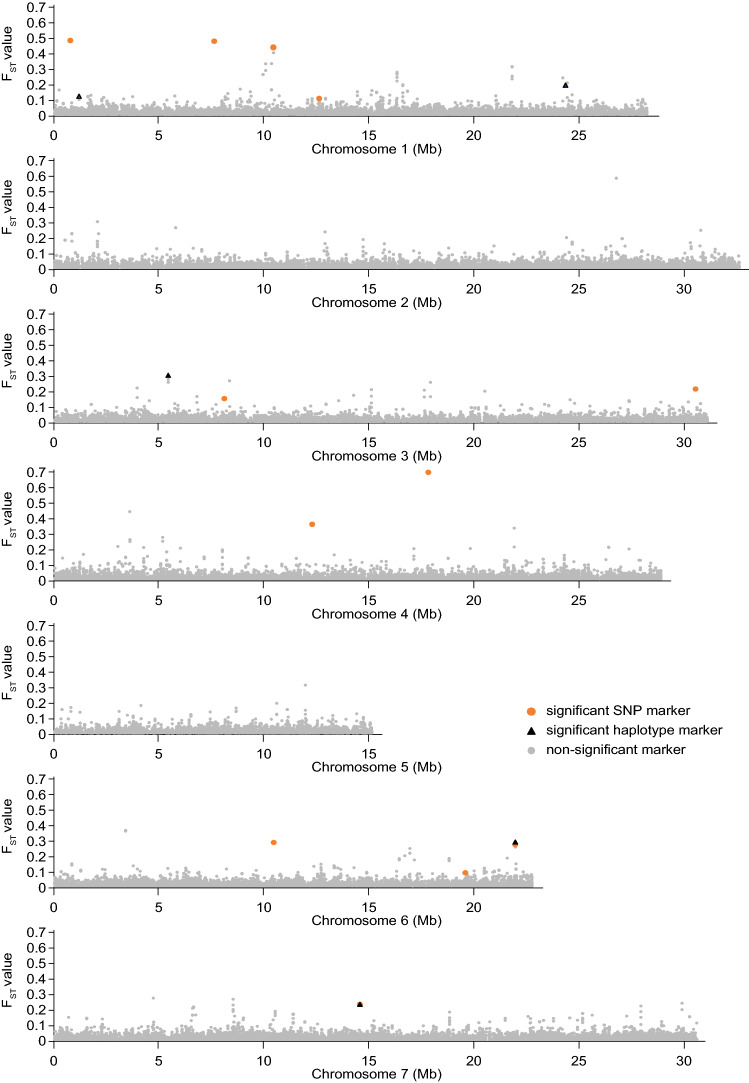
Single-nucleotide polymorphism and haplotype polymorphism with significantly different allele frequencies in early versus late elongating pools of red clover according to three different methods. All non-significant markers are also plotted. *X*-axes, chromosomal position (Mb); *y*-axes, *F*_ST_ value of the marker, based on allele frequencies in early versus late elongating pools

BayeScan detected a much higher number of markers with a significantly different allele frequency in the early elongating group versus the late elongating group than the two tests utilizing variation between replicates to evaluate significance. Among the latter, the F_ST_-based method (method 1) detected a higher number than the method based on error variance (method 2) (Supplementary File 2). Across marker types, we detected a total of 20 loci that were significant according to all three methods. Of these, 15 had a known chromosomal location; 10 were detected based on the *Pst*I data set and 5 on the *Ape*KI data set (Table 1). Ten of the 15 loci were found only in the SNP analyses, three only in the haplotype analyses, while only two loci were found both among SNPs and haplotypes. The 15 loci were distributed across all chromosomes except chromosome 2 and chromosome 5, with 1–6 loci per chromosome (Fig. 3). The maximum calculated F_ST_ value of markers within significant chromosomal loci ranged from 0.08 to 0.68.

**Table 1:** Loci with significantly different marker allele frequencies in early and late phenotypic groups of red clover, cultivar ‘Lea’, according to all of three different tests (BayeScan and two tests utilizing replicate pools) at a false discovery rate of 0.05

Locus^4^	SNPs^1^			HTPs^2^			
	Position	Allele frequency (RAF^3^)		Position^5^	Haplotypes^6^	Allele frequency	
		Early	Late			Early	Late
1_0.76 (*Pst*I)	760722	0.27	0.95				
1_1.16 (*Pst*I)				1162890–1162976/ +	000000	0.37	0.70
1_7.60 (*Pst*I)	7596707	0.84	1.00				
1_10.40 (*Pst*I)	10404396	0.33	0.95				
	10404400	0.32	0.95				
	10404420	0.33	0.95				
	10404428	0.33	0.95				
1_12.58 (*Pst*I)	12578823	1.00	0.82				
1_24.28 (*Ape*KI)				24282619–24282693/ +	00 001	0.90 0.09	0.51 0.46
3_5.42 (*Ape*KI)				5423909–5423983/−	000 010	0.83 0.17	0.30 0.70
3_8.10 (*Ape*KI)	8098050	0.43	0.80				
3_30.48 (*Pst*I)	30484514	0.14	0.58				
4_12.26 (*Pst*I)	12255468	1.00	0.48				
4_17.77 (*Pst*I)	17771667	0.96	0.14				
6_10.41 (*Ape*KI)	10405305	1.00	0.56				
6_19.51 (*Pst*I)	19510781	0.85	1.00				
6_21.88 (*Ape*KI)	21881050	0.38	0.88	21881007–21881081/ +	000 010	0.38 0.62	0.89 0.11
7_14.52 (*Pst*I)	14523935	1.00	0.63	14523913–14523999/ +	0000	1.00	0.63
	14523937	1.00	0.63		0110	0.00	0.37

^1^Single-nucleotide polymorphism; ^2^Haplotype polymorphism; ^3^Reference allele sequence; ^4^Chromosome number_region (Mb); ^5^/ + , sequenced in the direction of the reference genome; /−, sequenced in the reverse direction; ^6^coding of stack mapping anchor point polymorphisms (SMAPs) and SNPs of significant haplotypes (non-significant haplotypes are not shown), where 0 indicates similarity to the reference genome, 1 indicates a difference, and “.” indicates an SMAP

The 199 genes present in the ± 50 kb regions flanking the 15 loci are reported in Supplementary File 3; these are candidate genes that may contribute to the differentiation between early and late elongating groups. Within 13 of these regions, we identified a number of genes that, based on their functional annotation and similarity to genes with known functions in *A. thaliana* or *M. truncatula*, have potential roles in the vernalization, autonomous and photoperiod regulation of floral transition, as well as hormonal and cellular signaling of stem elongation and cell growth. (Table 2 and Supplementary File 4).

**Table 2:** Selected candidate genes present in the ± 50 kb regions flanking markers with significantly different allele frequencies in early versus late elongating pools of red clover

Candidate gene(s)	Locus	Putative pathway/process	Putative function
Frigida-like protein (FRL)	1_1.16	Vernalization	Transcription factor
Antagonist of like heterochromatin protein 1 (ALP1)	4_17.77	Vernalization/autonomous	Inhibition of PRC2-mediated silencing of FT-like genes
B3 domain-containing REM protein	1_10.40 (3) 1_24.28 (2)	Vernalization/meristem identity/organogenesis	Transcription factors
RNA-directed DNA methylation	1_0.76 7_14.52	Regulating timing of reproductive development	Transcriptional gene silencing
Dicer-like 2 (DCL2)	3_30.48	Regulating development	Transcriptional and post-transcriptional gene silencing
DExH-box ATP-dependent RNA helicases	1_12.58	Regulating timing of reproductive development	Post-transcriptional gene silencing RNA processing
Ribosome production factor 1	1_7.60	Regulating timing of reproductive development	RNA processing
Receptor-like kinases similar to FERONIA	6_21.88 (2)	Vernalization/autonomous Photoperiod Cell elongation	Alternative splicing Interaction with circadian clock
Gibberellin insensitive 1 (GID1)	1_12.58	Gibberellin signaling	Gibberellin receptor, interaction with DELLAs
Indeterminate domain (IDD)	7_14.52	Gibberellin signaling Auxin signaling	Interaction with DELLAs Auxin synthesis and transport
Ovate family protein (OFP)	1_1.16	Organogenesis	Transcriptional repressor
Cyclin-dependent kinase (CdK) inhibitor	3_30.48 (2)	Cell cycle control	Binds and inhibits CdK
Auxin response factor (ARF)	7_14.52	Auxin signaling Stem growth Organ polarity	Transcriptional activator
Pectate lyase	1_0.76	Auxin response Loosening of cell walls and cell elongation	Pectin degradation
Cobra (COB) Cobra-like 4 (COB4)	6_21.88	Anisotropic cell elongation	Regulator of cellulose biogenesis and microfibril orientation
Fasciclin-like arabinoglucan proteins	7_14.52 (3)	Cell wall functioning	
Beta-fructofuranosidase	1_10.40	Cell wall formation	Provide hexoses for cellulose synthesis
UDP-arabinopyranose mutase 1	6_21.88	Cell wall formation	Provide UDP-arabinose for synthesis of pectin and hemicellulose
Sec14-like phosphatidylinositol transfer protein (SEC14L-PITP)	7_14.52	Auxin signaling Cellular trafficking	Phospholipid transfer between membranes
Pleiotropic drug resistance protein 1	6_10.41 (6)	Strigolactone signaling Cell to cell transport	Transporter protein
Sorting nexin	1_10.40	Cellular trafficking	
Actin-related protein (ARP)	3_5.42	Cellular trafficking	Actin filament assembly and branching
Actin-depolymerizing factor (ADF)	3_8.10	Cellular trafficking	Actin cytoskeleton remodeling
Formin-like protein	4_17.77	Cellular trafficking	

More details are given in Table 1 and Supplementary Files 3 and 4

## Discussion

### Marker density and linkage disequilibrium

Identification of genomic regions and candidates for genes that control the transition to reproductive development in red clover using GBS relies on the density of polymorphic read loci, and the linkage disequilibrium (LD) between these loci and the allelic variation that controls the phenotypic trait. If LD decays at short distances, the required marker density is high. The level of LD in a synthetic variety depends on the number of parents and the relatedness between them (i.e., the level of genetic diversity) as well as the number of generations after the original crossing between these parents (Rafalski, 2002; Auzanneau et al. 2007). “Lea” is a synthetic variety made from 33 parental genotypes (15 individuals of the Norwegian variety “Nordi”, 7 individuals from the Swedish variety/landrace “Bjursele”, and 11 individuals from a Norwegian synthetic population (LGRk 8801−2× = Syn 1/88 2×, Vestad 1990), *pers. comm*. Petter Marum, Graminor AS, Norway). Commercial seed was used in the present study; the exact number of generations is not known, but is likely to be around 4 and therefore the chromosomal segments inherited from the 33 parents are likely to be rather long. However, with this many parents, there are theoretically up to 66 different haplotypes present per chromosomal segment. De Vega et al. (2015) characterized the same seed lot as the one we used in the present study and found that the average LD (scaled from 0 to 1) at distances of 100 kb, ranged between 0.19 and 0.25 for the different chromosomes. At 500 kb, LD had decayed completely to background levels (0.02–0.05). In the present study, the average distance on the reference genome between polymorphic GBS loci was 5.4 kb, which appears to be sufficient to detect a large proportion of loci underlying the phenotypic differentiation, provided that the effect on the phenotype and the power of the statistical tests are strong enough.

Sequence variation in genes in the proximity of markers with significantly different allele frequency in early versus late pools potentially contributes to phenotypic variation in earliness of stem elongation. Based on a previous study of the LD decay in the same population (De Vega et al. 2015), we considered any gene within 50 kb flanking a significant marker as a candidate gene. Based on currently available knowledge about regulation of stem elongation and the transition to reproductive development in plants in general, we here discuss the possible role of the apparently most relevant candidate genes.

### Regulation of transition to reproductive development

In a previous study of vernalized plants of three Norwegian cultivars, including Lea, which we used here, we found that the effect of ambient temperature on number of days to stem elongation leveled off between 10 and 14 °C (at both 16 and 20 h photoperiod; Ergon et al. 2016). DTE decreased as photoperiod increased from 16 to 20 h (at temperatures between 14 and 18 °C). In the current study, plants were grown in a greenhouse at 16 °C, with a 20 h photoperiod, and although these plants were not vernalized, we regard it likely that the ambient temperature requirement was saturated, while the photoperiod requirement might not have been. Although red clover generally has very little requirement for vernalization, some Norwegian material responds to vernalization by flowering earlier (Van Dobben 1964). Hence, we consider that the variation in timing of stem elongation that we observed within cultivar Lea in the present study is likely reflecting variation in photoperiod requirement, vernalization requirement and/or juvenility, and thus that the identified candidate genes flanking the significant markers may be involved in corresponding pathways controlling reproductive development in red clover.

Several of the identified candidate genes have potential roles in the autonomous or vernalization pathway regulating transition from vegetative to reproductive development (Table 2). A vernalization response has evolved independently many times and regulatory pathways differ among different angiosperm lineages (Bouché et al. 2017). A central gene in the vernalization response of *A. thaliana* is *FLOWERING LOCUS C (FLC).* FRIGIDA (FRI) is an activator of *FLC* expression*,* while several genes in the autonomous and vernalization pathways are repressors of *FLC*. FRI overrides the effect of components of the autonomous pathway and thereby creates a requirement for vernalization, and vernalization overrides this effect of FRI. Orthologs of *FRI* and *FLC* do not appear to be present in the legumes studied so far (Kim et al. 2013), but some *FRI*-like genes are identified. One of the candidate genes we identified, *Tripr.gene5544,* at locus 1_1.16, is an ortholog of *M. truncatula* Medtr1g103710, encoding an FRI-like protein and located in a syntenic region of chromosome 1. *Tripr.gene5544* has almost 100% identity at mRNA level with an *FRI-*like gene in *M. sativa*, *FRI-L,* which was identified by Chao et al. (2013). Expression of MsFRI-L in transgenic *A. thaliana* plants resulted in late flowering phenotypes. In addition, transcript profiling of floral regulatory genes in these transgenic plants showed enhanced expression of the flowering repressor *FLC* and decreased expression of *FT,* suggesting that *MsFRI-L* delays flowering time by regulating gene expression (Chao et al. 2013). This supports a role of *Tripr.gene5544* in control of transition to reproductive development in red clover. However, as *FLC* appears not to be present in legumes, the molecular function of both *MsFRI-L* and *Tripr.gene5544* remains unknown.

A candidate at locus 4_17.77 is similar to *ANTAGONIST OF LIKE HETEROCHROMATIN PROTEIN 1* (*ALP1)*, another gene that regulates *FLC* in *A. thaliana*. ALP1 is a PIF/Harbinger class transposase that has acquired a novel function in epigenetic gene regulation in the plant kingdom (Liang et al. 2015). ALP1 inhibits polycomb group (PcG)-mediated *FLC* silencing in *A. thaliana* by blocking the interaction of the core REDUCED VERNALIZATION RESPONSE 2—Polycomb repressor complex 2 (VRN2-PRC2) with some of its accessory components. PRC2 takes part in epigenetic regulation not only of FLC but of a range of genes, e.g., the central flowering signal gene *FT* (Jiang et al. 2008), meaning that ALP1 could possibly affect timing of transition to reproduction through genes other than *FLC.*

Two of the loci that we identified (1_10.40 and 1_24.28) harbor in total 5 genes belonging to the REM family of B3 DNA-binding domain proteins. This family comprises 45 members in *A. thaliana* (Romanel et al. 2009), of which some are known to be involved in the vernalization pathway, e.g., *VERNALIZATION1* (*VRN1*). Several other REM genes are involved in inflorescence meristem identity or development of flowers (Mantegazza et al. 2014). The REM family has experienced a rapid divergence in plants (Romanel et al. 2009). For example, Verma and Bhatia (2019) identified 19 REM genes in *Cicer arietinum*, of which they found only 3 homologs in *M. truncatula* and none in *Glycine max* or *A. thaliana*. Hence, it is difficult to identify orthologs and to hypothesize about the possible specific roles of the *REM* genes that we identified near significant markers.

In addition to the flowering-related transcription factors mentioned above, we found several genes belonging to families that are more generally known to be involved in transcriptional or post-transcriptional gene regulation. At least five of the identified loci (1_0.76, 1_7.60, 1_12.58, 3_30.48 and 7_14.52) contained genes encoding proteins with putative roles in RNA processing, ribosome biogenesis and transcriptional or post-transcriptional gene silencing (two RNA-directed DNA methylation, a Dicer-like (DCL) gene, two DExH-box ATP-dependent RNA helicases and ribosome production factor (RPF)). RDM and DCL genes are involved in the RNA-directed DNA methylation (RdDM) pathway of transcriptional gene silencing (TGS) (Rowley et al. 2011; Matzke and Mosher 2014; Borges and Martienssen 2015). RdDM is the major small interfering RNA (siRNA)-mediated epigenetic silencing mechanism in plants and it has a range of biological roles (Matzke and Mosher 2014). Some RDM proteins and DExH RNA helicases can regulate flowering time through RNA silencing of genes in the autonomous pathway (Herr et al. 2006; Greenberg et al. 2011) and some RPF genes can affect flowering time or stem length (Weis et al. 2015; Maekawa et al. 2018; Choi et al. 2020).

There are a large number of receptor-like kinases in plants, of which some have been shown to play developmental roles (Wang et al. 2008). One of the identified loci (6_21.88) includes two genes with some similarity to the cell wall-associated receptor-like kinases *FERONIA (FER)*, which takes part in the photoperiodic, vernalization and autonomous signaling pathways of floral transition and a range of other processes in *A. thaliana* (Wang et al. 2020a; Solis-Miranda et al. 2020). FER can also regulate cell elongation or cell wall formation (Galindo-Trigo et al. 2016; Li et al. 2017). FER belongs to the CrRLKL1 subfamily of receptor-like kinases that has 36 members in *M. truncatula*, but the function of the individual genes is not known (Solis-Miranda et al. 2020).

In some of the regions flanking significant markers, we found genes related to hormonal signaling. Active forms of gibberellic acid (GA) stimulate stem elongation in legumes like pea and *M. truncatula* (Reinecke et al. 2013; Jaudal et al. 2018), but are thought to be of lesser importance for floral transition in legumes than in *A. thaliana* and were found to either not affect or delay flowering in pea (Weller et al. 1997; Reinecke et al. 2013; Liew et al. 2014). Both GA and auxin have regulatory functions in cell division and subsequent cell elongation in the shoot apex and the developing stem (Serrano-Mislata and Sablowski 2018; McKim 2019, 2020). In red clover, buds on axillary branches give rise to elongating flowering stems, and the main shoot does not. Hence, apical dominance resulting from auxin repression of axillary bud outgrowth, via strigolactone and cytokinin (Barbier et al. 2019), must be broken.

The promoting effect of GA on both flowering and stem elongation in *A. thaliana* is mainly accomplished through their interaction with DELLA proteins (Hedden and Sponsel 2015; Bao et al. 2020). When bound to GA, GID1 proteins bind DELLA proteins and target them for destruction by the 26S proteasome. This removes the suppressive action of DELLA on a range of factors promoting growth and development. In addition to this proteolytic mechanism, GID1 proteins are involved in interactions between GA and DELLA that affect growth and development through non-proteolytic mechanisms (Hauvermale et al. 2014). GID1 and DELLA are also present and interact in *M. truncatula* (Jiao et al. 2020). One of the candidates that we identified was a gene similar to *M. truncatula GIBBERELIN-INSENSITIVE 1C* (GID1C) (Wang et al. 2020b)*,* located in 1_12.58. A gene similar to the *INDETERMINATE DOMAIN (IDD)* group of the C2H2 zinc finger protein family was located in 7_14.52. There are at least 19 *IDD*-like genes in *M. truncatula* (Jiao et al. 2020). IDD proteins can function in floral transition and a variety of other processes; some can compete in binding to DELLA proteins, and thereby regulate growth and development, while others can regulate auxin synthesis and transport (Kumar et al. 2019). An *OVATE FAMILY PROTEIN* (*OFP*) was located in 1_1.16*.* OFPs are transcriptional repressors that regulate multiple aspects of plant growth and development, which are likely achieved by interaction with different types of transcription factors and/or by directly regulating the expression of target genes such as *Gibberellin 20 oxidase* (*GA20ox*) (Wang et al. 2016). Some delay flowering and inhibit stem growth (Wang et al. 2016; Zhang et al. 2018). Finally, we found two cyclin-dependent kinase (CdK) inhibitors (locus 3_30.48), which can bind to CdKs and thus control cell cycle progression in interaction with abscisic acid, cytokinin and GA (Francis and Sorrell 2001).

AUXIN RESPONSE FACTORs (ARFs), of which we found one in locus 7_14.52, are transcription factors mediating auxin-induced gene expression (Die et al. 2018; Gao et al. 2019; Gomes and Scortecci 2021). One effect of auxin is its stimulation of cell elongation through transcriptional changes leading to increased cell wall acidification and extensibility, allowing for cell elongation (Arsuffi and Braybrook, 2018; Majda and Robert 2018; Wang et al. 2020b). This is achieved partly through the upregulation of cell wall-modifying enzymes, like for example pectate lyase (locus 1_0.76), which is regulated by auxin, degrades pectin and thus contributes to increased cell wall extensibility. Candidate genes with roles in cell wall formation were also found. Both COBRA (COB) (locus 6_21.88) and FASCICLIN-LIKE ARABINOGLUCAN PROTEINs (FLA) (locus 7_14.52) are glucosylphosphatidylinositol (GPI)-anchored proteins (Roudier et al. 2005; Schultz et al. 2002; Huang et al. 2013; He et al. 2019). COB proteins are thought to control anisotropic cell expansion through their involvement with the orientation of cellulose microfibrils transversely to the axis of elongation (Roudier et al. 2005), while *FLA* proteins have roles in for example secondary cell wall formation. Some of our significant markers are located near genes involved in providing the building blocks for the synthesis of cellulose, hemicellulose and pectin in cell walls (a beta-fructofurantosidase, locus 1_10.40, and a UDP-arabinopyranose mutase, locus 6_21.88). Sucrose, transported from photosynthetic or storage tissues through the phloem, can be hydrolyzed by either sucrose synthases or invertases, and the latter has been shown to be responsible for making hexoses available for cellulose synthesis, as invertase mutants, but not sucrose synthase mutants, strongly reduce growth in both *Lotus japonicus* and *A. thaliana* (Welham et al. 2009; Barratt et al. 2009). L-arabinose, found in pectins and hemicelluloses, is derived from cell wall UDP-arabinofuranose, which is converted from cytosolic UDP-arabinopyranose by UDP-arabinopyranose mutases (Saqib et al. 2019).

Both signaling and the cell wall formation itself depend on trafficking of signaling molecules and compounds used as building blocks through the cellular membrane systems. Locus 6_10.41 harbors six copies of *PLEIOTROPIC DRUG RESISTANCE PROTEIN 1* (PDR1), which in petunia has been shown to be responsible for short-distance transport of strigolactone (Shiratake et al. 2019). In locus 7_14.52, we found a Sec14-like phosphatidylinositol transfer protein (SEC14L-PITP). The 35 SEC14L-PITPs in *A. thaliana* are associated with membrane systems and transfer different phospholipids (e.g., phosphatidylinositol) between membranes to stimulate signaling pathways leading to development and stress responses (Tejos et al. 2018; Zhou et al. 2019; Montag et al. 2020). Some SEC14L-PITPs are regulated by auxin (Tejos et al. 2018). SORTING NEXIN 1 (SNX1) (locus 1_10.40) is associated with a sorting endosome in *A. thaliana*, thought to play a role in cellular trafficking (Jaillais et al. 2008). Movement of membrane-bound vesicles in the cell is guided by the cytoskeleton, consisting of microtubules and actin filaments. Among the identified candidate genes are *ACTIN-RELATED PROTEIN 3/DISTORTED 1 *(ARP3/DIS1), an *ACTIN DEPOLYMERIZATION FACTOR *(ADF) and an *FORMIN-*like gene (locus 3_5.42, 3_8.10 and 4_17.77, respectively)*.* These proteins play key roles in remodeling and function of actin (Kandasamy et al. 2004; Staiger and Blanchoin 2006; Nan et al. 2017). ARP3/DIS1 has also been shown to play a role in PIN-mediated polar auxin transport in *A. thaliana* root cells (Zou et al. 2016).

Finally, several other genes in the significant loci encode proteins with regulatory functions and which may be involved in developmental processes, e.g., F-box/LRR proteins, protein phosphatases, ribosomal and RNA-binding proteins, zinc finger proteins, pentatricopeptide proteins, ubiquitins, transmembrane proteins, WD40-repeat proteins, calcium-binding proteins, nodulin-like proteins, myb transcription factors, and methyltransferases.

### Conclusions

Performing GBS on replicate pools of phenotypic extremes in a population allowed us to identify genetic markers with significantly different allele frequencies in the two phenotypic groups, in this case red clover with early and late stem elongation. SNPs and GBS locus haplotypes were complementary, so that using both allowed us to identify a higher number of significant loci. Given the low LD in the studied population, candidates for genes controlling the trait can be assumed to be relatively close to the marker. Within ± 50 kb regions flanking significant markers, we found genes with potential roles in the vernalization, autonomous, and photoperiod regulation of floral transition, hormonal regulation of stem elongation and cell growth. These results provide a first insight into the potential genes and mechanisms controlling transition to stem elongation in a forage legume.

## Supplementary Information

Below is the link to the electronic supplementary material.

Supplementary file1 (DOCX 18 KB)

Supplementary file2 (DOCX 18 KB)

Supplementary file3 (XLSX 36 KB)

Supplementary file4 (XLSX 24 KB)

## Declarations

### Data availability

The raw sequence data split per DNA pool (three early, three late) per library preparation method (*Pst*I, *Ape*KI) are available in NCBI SRA under accession number PRJNA784180. The SNP and haplotype allele frequencies per pool are deposited in the NMBU Open Research Data database (https://doi.org/10.18710/P6FYU7).
