# Supplementary material for: Identification of loci controlling timing of stem elongation in red clover using genotyping by sequencing of pooled phenotypic extremes
Source: Mol Genet Genomics. 2022 Aug 24;297(6):1587–600. doi: 10.1007/s00438-022-01942-x (PMC9596541; doi:10.1007/s00438-022-01942-x)
Supplement: Supplementary file 1 — Supplementary file1 (DOCX 18 KB) [file 438_2022_1942_MOESM1_ESM.docx]

Identification of loci controlling timing of stem elongation in red clover using genotyping by sequencing of pooled phenotypic extremes

*Molecular Genetics and Genomics*

Åshild Ergon*, Øystein W. Milvang, Leif Skøt, Tom Ruttink
*Dept. of Plant Sciences, Faculty of Biosciences, Norwegian University of Life Sciences. ashild.ergon@nmbu.no

**Supplementary File 1.** Number of single nucleotide polymorphisms (SNPs) and haplotype polymorphisms (HTPs) obtained from genotyping by sequencing of libraries prepared from pooled DNA samples of red clover individuals, using two different restriction enzymes. The number of markers and loci with a known chromosomal location is given, with additional markers and loci on unplaced scaffolds in parentheses.

|  | Restriction enzyme | |
| --- | --- | --- |
|  | *Pst*I | *Ape*KI |
| Number of SNPs after filtering^1^ | 8051 (4023) | 58407 (32729) |
| Number of haplotypes in polymorphic GBS loci (HTPs) | 10867 (5272) | 95911 (50133) |
| Number of HTPs^3^ after filtering^2^ | 3029 (1448) | 27111 (14027) |
| Average number of haplotypes per HTP (range) | 3.6 (2-13) | 3.6 (2-20) |

^1^Only SNPs and HTPs with minimum 30 reads in each of the six pools and minor allele frequency > 0.05 in at least one pool were kept. ^2^Haplotype polymorphisms, defined by SNPs and/or stack anchor mapping points (SMAPs) in read stacks.
