# Supplementary material for: Identification of loci controlling timing of stem elongation in red clover using genotyping by sequencing of pooled phenotypic extremes
Source: Mol Genet Genomics. 2022 Aug 24;297(6):1587–600. doi: 10.1007/s00438-022-01942-x (PMC9596541; doi:10.1007/s00438-022-01942-x)
Supplement: Supplementary file 2 — Supplementary file2 (DOCX 18 KB) [file 438_2022_1942_MOESM2_ESM.docx]

Identification of loci controlling timing of stem elongation in red clover using genotyping by sequencing of pooled phenotypic extremes

*Molecular Genetics and Genomics*

Åshild Ergon*, Øystein W. Milvang, Leif Skøt, Tom Ruttink
*Dept. of Plant Sciences, Faculty of Biosciences, Norwegian University of Life Sciences. ashild.ergon@nmbu.no

**Supplementary File 2.** Total number of SNPs (A) and haplotype polymorphisms (HTPs) (B) with different allele frequency in early versus late groups of red clover individuals. The number of loci that these markers represent is also given. The number of markers and loci with a known chromosomal location is given, with additional markers and loci on unplaced scaffolds in parentheses. The marker data were obtained from genotyping by sequencing libraries of pooled DNA samples prepared using either *Pst*I or *Ape*KI as the restriction enzyme. BayeScan as well as two tests utilizing replicate pools, based on F_ST_-values (method 1) or error variances (method 2), were used to identify markers with different allele frequency at a false discovery rate of 0.05.

(A)

|  | *Pst*I |  | *Ape*KI |  |
| --- | --- | --- | --- | --- |
|  | SNPs | Loci | SNPs | Loci |
| Method 1 | 28 (14) | 18 (10) | 34 (20) | 29 (16) |
| Method 2 | 13 (2) | 9 (2) | 3 (4) | 3 (3) |
| BayeScan | 80 (58) | 47 (30) | 201 (125) | 158 (98) |
| In common | 13 (2) | 9 (2) | 3 (3) | 3 (2) |

(B)

|  | *Pst*I |  | *Ape*KI |  |
| --- | --- | --- | --- | --- |
|  | Haplotypes | HTPs | Haplotypes | HTPs |
| Method 1 | 12 (11) | 10 (7) | 45 (31) | 38 (26) |
| Method 2 | 3 (0) | 2 (0) | 6 (3) | 3 (3) |
| BayeScan | 80 (54) | 79 (50) | 374 (234) | 335 (204) |
| In common | 3 (0) | 2 (0) | 3 (3) | 3 (3) |
